# Associational resistance through intercropping reduces yield losses to soil‐borne pests and diseases

**DOI:** 10.1111/nph.18302

**Published:** 2022-07-01

**Authors:** Victoria G. A. Chadfield, Sue E. Hartley, Kelly R. Redeker

**Affiliations:** ^1^ Department of Biology University of York Wentworth Way York YO10 5DD UK

**Keywords:** agroecology, associational resistance, co‐crops, ecological interactions, meta‐analysis, meta‐regression, nematodes, pathogen

## Abstract

- Associational resistance to herbivore and pathogen attack is a well documented ecological phenomenon and, if applied to agriculture, may reduce impact of pests and diseases on crop yields without recourse to pesticides.
- The value of associational resistance through intercropping, planting multiple crops alongside each other, as a sustainable control method remains unclear, due to variable outcomes reported in the published literature. We performed a meta‐analysis to provide a quantitative assessment of benefits of intercropping for target plant resistance to plant‐parasitic nematodes and soil‐borne diseases.
- We found that intercropping reduced damage to focal crops from nematodes by 40% and disease incidence by 55%. Intercropping efficacy varied with biological variables, such as field fertilisation status and intercrop family, and methodology, including whether study samples were potted or in fields.
- Nematode control using intercropping was sufficient to offset reductions in focal crop yield from intercrop presence, making intercropping a viable agricultural tool. We identify key drivers for underpinning the success of intercropping and indicate areas for future research to improve efficacy. This study also highlights the potential benefits of harnessing ecological knowledge on plant–enemy interactions for improving agricultural and landscape sustainability.

## Introduction

Plants are at the centre of a complex network of interactions, including those with competing individuals and species, as well as natural enemies, both above and below ground (Johnson *et al*., 2016). The nature of these interactions between plants and their herbivores and pathogens is altered by close‐proximity neighbouring plants, particularly for heterospecifics (Tahvanainen & Root, 1972). Associational resistance is a recognised, widespread ecological interaction whereby specific plant associations with unpalatable neighbours decrease the likelihood of detection by, and vulnerability to, herbivore attack; in other cases, known as associational susceptibility, interactions with neighbouring plants lead to an increase in such attacks (Barbosa *et al*., 2009).

Ecological intensification, the practice of utilising natural ecological processes to replace intensive anthropogenic inputs in agricultural systems, has been proposed as a means to increase the environmental sustainability of agriculture (Bommarco *et al*., 2013). Future changes in climate, population and patterns of consumption are predicted to require an increase over current crop production of 100% by 2050 (Tilman *et al*., 2011), which clearly cannot be delivered sustainably by devoting more land to agriculture or by increasing already high levels of external inputs. Crop losses due to weeds, insects, nematodes and pathogens exacerbate this problem, standing at 26–40%, even with the best current control methods (Oerke & Dehne, 2004; Oerke, 2006). Furthermore, these losses are predicted to increase under climate change (Deutsch *et al*., 2018). The application of associational resistance approaches could be a sustainable method to address some of the current and are likely to be future crop losses to pests.

Much research on the influence of close neighbours to plant resistance has focused on aboveground herbivores in natural systems, but plants come under attack from many other types of enemies, especially in the monocultures and high input environments of intensive agriculture. Plant natural enemies in soil, such as plant‐parasitic nematodes and soil‐borne diseases, reduce the yields of many crops worldwide (Shigaki *et al*., 1998; Oerke & Dehne, 2004; Marimuthu *et al*., 2013) and are particularly difficult to control through pesticide application (Matthiessen & Kirkegaard, 2006). Some pathogenic organisms, such as potato cyst nematodes (*Globodera* spp.), can persist in soil in a resistant state for decades (Evans & Stone, 1977), quiescent until soil pesticide concentrations are reduced to ineffective levels. Adsorption and degradation of pesticides in agricultural soils can prevent sufficient exposure for control (Munnecke, 1972), particularly in the context of restrictions on their use due to the impacts on environmental and human health (Popp *et al*., 2013). There is an urgent need for alternatives to current practice, alternatives based on approaches that apply a knowledge of plant ecology and biology to improve food crop production sustainably (Hartley, 2018; Pretty *et al*., 2018). Many of these approaches, such as intercropping, in which more than one plant species is cultivated in the same plot at the same time, do not require major or technically difficult advances so could be implemented quickly, and with more research into underpinning ecological mechanisms they could be more widely adopted (Royal Society, 2009).

Some planting regimes can diversify agricultural systems, including temporal diversification through expanded rotations to include cover crops or spatial diversification via cultivar mixing and intercropping (Reiss & Drinkwater, 2018). Of these potential options, intercropping has been identified as a potential sustainable strategy for maximising crop yields while combating pest damage and disease, including those caused by soil‐borne organisms (Malézieux *et al*., 2009; Boudreau, 2013). Intercropping, applying the framework of associational resistance, may influence pest and disease damage through (1) modifying soil nutrient and water availability, (2) interfering with pest capacity to detect/recognise focal crop root structures, (3) inducing defences in focal crops through chemical cues released by the intercrop, and (4) provision of habitat for known natural enemies of focal crop pests (Barbosa *et al*., 2009). Associational susceptibility is driven by the converse of these functional behaviours.

Studies of the value of intercropping for sustainable pest control have found mixed and sometimes conflicting results, reflecting the complex and variable nature of associational resistance and susceptibility. For example, intercropping cucumber with hot pepper, castor or crown daisy reduced root knot nematode (*Meloidogyne incognita*) attack, whereas damage increased with hairy vetch, common zinnia, and Baikal skullcap intercrops (Dong *et al*., 2012). Intercropping can also lead to variable outcomes for focal crop diseases. For example, anise and garlic intercrops reduced damping‐off and root rot in lentil regardless of whether the causal pathogen was *Rhizoctonia solani* or *Fusarium solani*, whereas intercropping with onion only protected lentil from *R. solani* (Abdel‐Monaim & Abo‐Elyousr, 2012).

This variability in published results is perhaps unsurprising given that intercrops can affect nematode damage or disease incidence through a range of mechanisms that, combined with the complex nature of plant–soil interactions in agroecosystems, is likely to cause significant variability in outcomes. These mechanisms can be broadly divided into three categories (following Trenbath, 1993): (1) direct impacts on the survival, activity, or reproduction of the pest/pathogen; (2) indirect impacts on the pest/pathogen via changes in the focal plant species; and (3) indirect impacts on the pest‐pathogen via changes in the soil community (including natural enemies) or in the soil environment. Given the conflicting but burgeoning literature, a robust quantitative statistical review of the effects of intercropping on plant‐parasitic nematode damage and on soil‐borne disease incidence is needed and timely but has not been attempted to date. This sort of contingency is a barrier to uptake of sustainable approaches in agriculture systems (Doheny‐Adams *et al*., 2018; Hartley, 2018), so a better understanding of the factors driving variability in outcomes is vital.

Our study was designed to test whether intercropping is an effective agricultural tool to reduce nematode and pathogen impacts on focal crop yields. We addressed this knowledge gap using meta‐analysis, an approach that has been used successfully to synthesise results from studies in field environments (e.g. Denno *et al*., 2008; Letourneau *et al*., 2011; Johnson *et al*., 2012). Specifically, we used meta‐analysis, combined with meta‐regression, to investigate mechanisms underpinning the relationship between intercropping and damage to focal crops from parasitic nematodes or soil‐borne diseases. The role of any given mechanism will be context‐dependent, and we explored the influence of these contextual factors through meta‐regression, a technique that quantifies the heterogeneity between experimental outcomes by fitting a range of moderator variables (Berkey *et al*., 1995).

Considering the mechanisms of action for associational resistance, we hypothesised that resource availability, plant density/diversity as well as both intercrop and pathogen species identity/guild are primary drivers of interactions between intercrop plants, focal crop plants and herbivores/diseases in agricultural systems. For instance, we might expect greater intercrop planting density to create a more challenging ‘maze’, making it more difficult for pests to detect and move to focal crop plant roots. Similarly, intercrop plants known to produce bioactive compounds may be more likely to be effective in conferring associational resistance. At this time, exact ecological mechanisms of control remain contested in ecosystems displaying associational resistance (Agrawal *et al*., 2006), so these parameters were targeted to provide insight into underlying mechanisms and to determine the effectiveness of intercropping. We therefore tested the influence of a range of both biological and agronomic factors on damage or disease incidence in intercropped vs nonintercropped systems, including intercrop plant family, fertiliser inputs, pathogen inoculum source (i.e. laboratory cultured or field soil population), nematode species, pathogen type, timing of inoculation and intercrop addition, experiment duration and plant density. Furthermore, we tested whether the beneficial effects of intercropping on focal crop yield through reductions in the losses due to nematode damage or disease incidence were sufficient to offset yield losses due to the presence of the intercrop, so determining whether intercropping can be an environmentally and economically sustainable alternative to chemical control of soil‐borne pests and diseases.

## Description

### Literature search and study selection

We performed literature searches in the Web of Science (Core Collection; www.webofknowledge.com), the British Library (theses; www.explore.bl.uk), the Indian Citation Index (ICI; www.indiancitationindex.com), and the National Library of Australia Trove (theses and conference proceedings; www.trove.nla.gov.au) databases using the following topic keywords: intercrop* OR “inter‐crop*” OR cocrop* OR “co‐crop*” OR (mixed crop*) OR interplant* OR “inter‐plant*” OR bicrop* OR “bi‐crop” OR polycultur* OR “poly‐cultur*” OR dicultur* OR “di‐cultur*” OR (cover crop*) or companion* AND disease* OR patho* OR nemat*. Full search query strings are provided in supplementary information, under Supporting Information Notes S1). The final search of the Web of Science database was performed on 30 June 2020. The final searches in the British Library and Trove were performed by 11 June 2020, and the ICI was last searched on 4 August 2013. We did not place time limits on any of the searches, therefore studies from the full timespan in each database were searched: 1900–2020 for Web of Science; 1800–2020 for the British Library; 2004–2013 for ICI; unspecified to 2020 for Trove. We also used backwards and forwards citation following.

To be included, experiments were required to compare (1) damage caused by plant‐parasitic nematodes or disease incidence on a primary (focal) crop species grown alone as a monocrop (control) with (2) damage or incidence on the same focal crop when grown intercropped with one other plant species (treatment). Experiments had to be replicated (*n* ≥ 2) with randomly assigned treatments.

We contacted the author if a relevant paper did not report the data needed. When the necessary data was reported only in a figure, GraphGrabber v.1.5.5 (Dedross & Boardley, 2009) software was used to extract the values.

We found 52 studies containing data that met the relevance criteria for nematode damage, and calculated effect sizes for 326 experiments (Table S1). For soil‐borne disease, we found 28 studies that met the relevance criteria, yielding 117 effect sizes (Table S1).

### Effect size and variance

#### Metric choice

A common metric was needed to compare results from studies that used different variables, constructs or descriptors to measure nematode damage or disease incidence. We used response ratio (*R*), defined as the mean value for the damage/disease found within the treatment (intercrop) group divided by the mean value for the control (focal monocrop) group, as the effect size. *L*, defined as the natural logarithm of *R*, was used in the meta‐analysis calculations along with a nonparametric variance estimate that allowed more experiments to be included, giving greater statistical power (Mayerhofer *et al*., 2012). *L* was used because, unlike *R*, it responds to changes in the numerator or denominator equally and in a linear fashion. The sampling distribution of *L* is also more normal than that of *R* in small samples (Hedges *et al*., 1999). The equation for *L* is:

(Eqn 1) $$L=\log_{e}\;R=\log_{e}\left({\underline{x}}_{T}/{\underline{x}}_{C}\right)$$

where ${\underline{x}}_{T}$ is the mean value for the treatment (intercrop) group and ${\underline{x}}_{C}$ is the mean value for the control (focal monocrop) group (Hedges *et al*., 1999).

The equation for the nonparametric variance of *L* is:

(Eqn 2) $$v_{L}=\left(n_{T}+n_{C}\right)/\left(n_{T}n_{C}\right)$$

where $n_{T}$ is the sample size for the treatment group, and $n_{C}$ is the sample size for the control group (Mayerhofer *et al*., 2012).

#### Crop yield effect size

Using the yield of the focal crop in the monocrop control and intercrop treatment respectively, we calculated a yield effect size for each study that reported relevant yield data. Further analyses using meta‐regression (please refer to ‘Meta‐regressions’ in the Description section) explored whether intercropping‐related changes in damage are associated with yield. A summary analysis of the focal crop yield effect size was not performed, as the experiments collected were only a nonrepresentative subset of the available experiments on yield and intercropping in the presence of nematodes.

#### Response ratios with zeroes

The response ratio (*R*) could not be calculated for experiments in which the monocropping treatment had a damage or disease value of zero. To avoid introducing bias against experiments in which the focal monocrop control had no observed damage or disease but the intercrop treatment did, we used the highest value of *L* in the final dataset in place of the infinitely large effect size that would have been calculated otherwise in these cases. Similarly, for experiments in which the intercropping treatment had a disease or damage value of zero (meaning *L* could not be calculated), we used the lowest value of *L* to avoid bias against experiments in which intercrop treatment reduced damage or disease below detectable levels.

### Experiment moderators

We collected details on experiment moderators for each experiment, including focal crop and intercrop species/density, water status, inoculum type and timing, as well as nematode species/lifestyle and/or pathogen genus (Table S1). We also described each experiment as either contained (in pots) or uncontained (in the ground, field‐based) and characterised the measurement construct utilised, whether these were direct measures or generalised indices (e.g. direct measures might include root damage/infection and/or plant mortality, whereas nematode/disease indices generalised individual plant results over a field area using a defined scale).

To ensure correct grouping, we used the current accepted binomial name for all species, using ‘The Plant List’,  database (www.theplantlist.org) to check plant species, and multiple sources to check nematode and pathogen species (Table S2). Table S2 also contains the references for the host status and susceptibility (of the intercrop in each experiment to the relevant nematode/pathogen) variables.

### Models

#### Summary random‐effects meta‐analyses

To allow generalisation of the results from this meta‐analysis we performed random‐effects meta‐analyses on the nematode and disease datasets when calculating the overall mean effect sizes (Hedges & Vevea, 1998). In addition, as intercropping is not expected to be identical in all agroecosystems, the random‐effects model is more appropriate because it does not assume that the true effect is identical in every experiment.

The estimated mean true effect sizes and estimated heterogeneity values were calculated using the *rma.mv* function in R in the metafor package (Viechtbauer, 2010). Each observed effect size was weighted by the inverse of its variance, with Experiment ID within Study ID included as nested random effects to include nonindependence of effect sizes from the same study and when multiple effect sizes were calculated using a shared experimental control or treatment.

#### Meta‐regressions

We used a mixed‐effects model for the meta‐regressions, retaining Experiment ID within Study ID as random effects but also with moderators as fixed effects, specified as a formula in the mods argument in the *rma.mv* function (Viechtbauer, 2010). For the heterogeneity estimator, we used maximum likelihood so that the model fit statistics could be compared during step‐wise model reduction. Base models, or meta‐regressions, were initially performed including moderators for which all available experiments provided data. These included nematode experiments from uncontained experiments (NU1; inclusive of ‘Measurement construct + Co‐crop family + Conditions + Water status + Fertilisation status’), nematode experiments in contained/potted experiments (NC1) and their disease counterparts (DU1, DC1) (Tables S3, S4). Meta‐regressions that initially included a moderator for which not every experiment in the full nematode or disease dataset had a reported value were run on subsets of the data so that those moderators could be investigated (e.g. NU3, for which nematode lifestyle was included along with all NU1 moderators) (Table S4). We also carried out summary meta‐analyses on each subset, to ascertain if the overall mean effect size was affected by the number of experiments included.

After running the initial model for a given subset, we removed the moderator with the highest nonsignificant *P*‐value and ran the reduced model. Results of the two models were compared using the *anova* function. We reduced the model step‐wise until all the moderators were significant or until reducing the model further explained a significantly lower amount of the heterogeneity in the data, according to the likelihood ratio test (Viechtbauer, 2010). If the moderator unique to that data subset dropped out, we abandoned the meta‐regression as the other moderators would already have been tested on a larger data subset (e.g. NU1, NC1, DU1 or DC1). All analyses were performed in R using the metafor package.

### Assessing bias

A ‘file drawer analysis’ was performed for the data(sub)set of each meta‐regression that yielded interesting results (and were therefore reported and discussed), to give the number of experiments (‘fail‐safe number’) with an average effect size of zero that would have to be added to the dataset to render the estimated mean true effect nonsignificant (Rosenthal, 1979). Funnel plots were also produced and examined for evidence of bias (Figs S1–S3). The file drawer analyses and funnel plots were performed in R using the metafor package.

## Results

The data set used across all meta‐analyses included 138 uncontained (in field) plus 188 contained (potted) nematode experiments and 89 uncontained plus 28 contained soil‐borne disease experiments. These published findings included 45 focal crops (limited due to the priority focus on soil pests and pathogens), 21 intercropping families, seven nematode genera inclusive of 16 nematode species, and 10 pathogen genera, including bacterial, fungal and oomycete varieties (Table S1). Beyond crop and pathogen type, other data from field and glasshouse trials were included as moderators (e.g. fertilisation and water status of the experiment, inoculum type (artificial or natural), and timing of intercrop planting relative to the focal crop) (Table S1). The combination of these moderators allowed us to determine which associational resistance mechanisms were most consistently effective within the agroecosystem context: (1) fertiliser and water status of the experiments allowed us to test the impact arising from modification of soil nutrient and water availability, (2) planting density and duration allowed us to examine the potential of physical barriers to interfere with pest capacity to detect/recognise the focal crop root structures, (3) intercrop family investigated the induction of defences in focal crops through chemical cues released by the intercrop, and (4) analysis of pest/pathogen genera tested the provision/reduction of habitat for focal crop pests/diseases and known natural enemies.

### Intercropping impacts on pest/disease damage reduction and implications for focal crop yield

Both disease incidence and pest damage were significantly reduced through intercropping. Soil‐borne disease damage to the focal crop within in‐field studies was reduced by 55% due to intercropping (with a 95% confidence interval (CI) of 67 to 38%) and 44% (95% CI of 57 to 28%) in contained experiments (Table S3). Nematode damage to the focal crop was reduced by 40% (95% CI of 55 to 20%) in uncontained, field‐based studies and 42% (95% CI of 54 to 28%) in contained, pot‐bound experiments (Table S3).

**Fig. 1:**
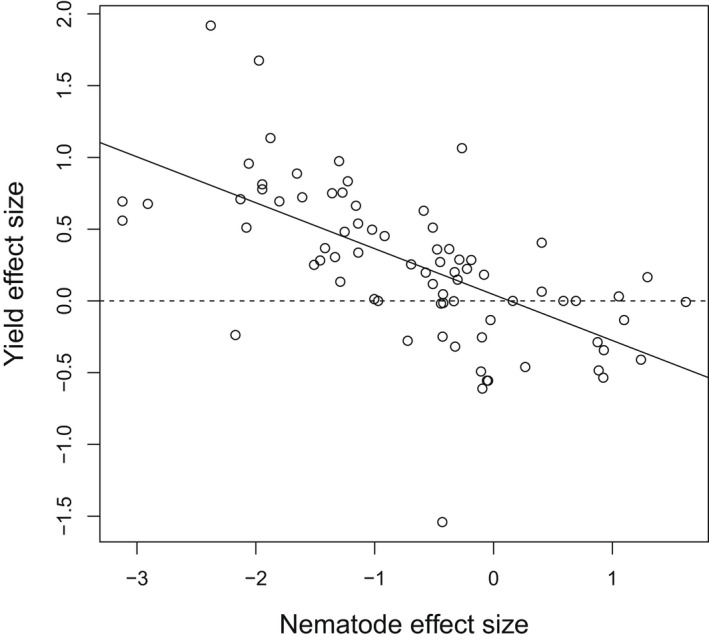
Plot of yield against nematode damage (both log‐response ratios where ±1.0 equals a deviation of 100%). The solid line is the regression line that corresponds to (log_e_(Yield response ratio) = − 0.320 × log_e_ (Damage response ratio) + 0.044. Slope confidence intervals (95%) are −0.476 to −0.164; while 95% CI for the intercept are −0.150 to 0.238. The dashed line (100% yield) marks where focal crop yield was the same in both monocrop and intercrop treatments. The solid regression line passes through 100% yield at a damage response ratio of 1.15. Assuming that the regression line is accurate, this suggests improving or maintaining focal crop yields in intercrops is possible even with an increase of up to 15% in focal crop damage under intercropping.

Nineteen studies that reported nematode damage data also contained yield information, for 76 experiments in total (and 11 studies, 66 experiments for disease; Table S1). The data published in these studies can be used to estimate how much damage reduction in intercropped fields is required to deliver equivalent focal crop yields to monocrops. Based on damage effect to focal crop yield regressions we estimate that anything more effective than a +15% enhancement in nematode damage (Fig. 1) results in improved focal crop yields from intercropped fields, compared with those obtained from monocrop planting. Whereas disease reduction is apparent in published studies, disease effect size was not significantly correlated to yield size and therefore estimates of reduced focal crop yield, necessary to obtain improved focal crop yield, were not possible.

### Identifying field and method variables that affect intercrop impact

**Fig. 2:**
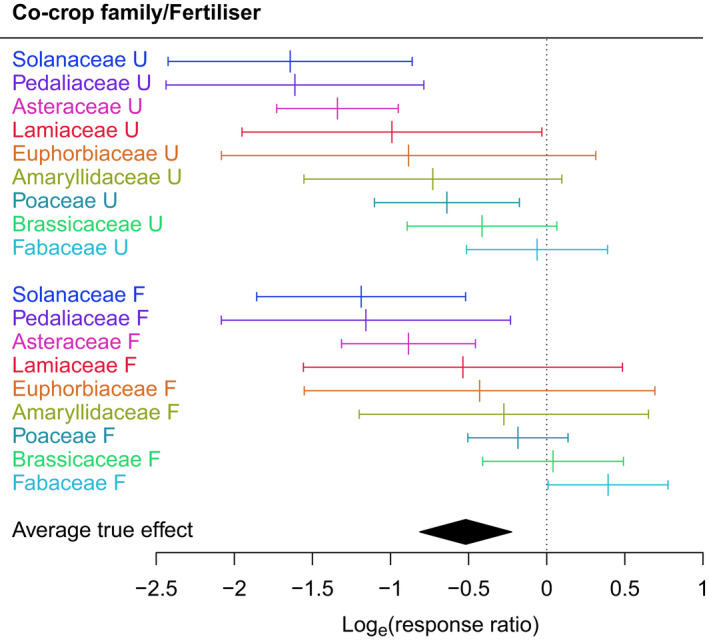
Predicted effects of intercropping on nematode damage with combinations of the moderators retained in the final model for the NU1 dataset, showing selected families (F, fertilised; U, unfertilised). Values below 1 show reduced nematode damage in intercrop studies. Error bars represent 95% confidence intervals.

The use of meta‐regressions allowed us to identify those experimental and field variables that were most influential. Within field‐based experiments the variables that were most influential in intercrop control of nematodes included intercrop family and field fertilisation status. Within the intercrop families most commonly reported, Asteraceae, Pedaliaceae and Solanaceae were found to be the most significantly effective in their capacity to moderate nematode damage (Fig. 2). Unfertilised fields showed significantly better outcomes (less nematode damage) compared with fertilised fields (Fig. 2).

**Fig. 3:**
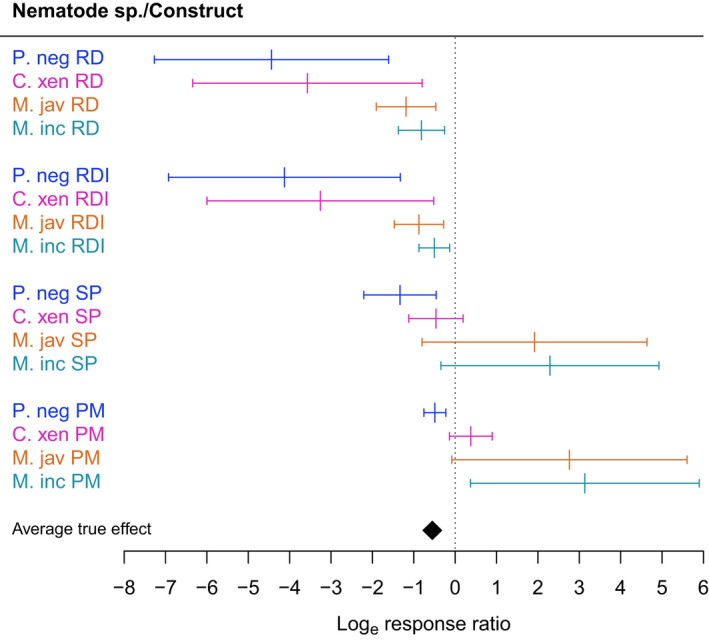
Predicted effects of intercropping on nematode damage with combinations of the moderators retained in the final model for the NC2 dataset, showing moderator categories most represented in the data. Measurement constructs and nematode species: RD, root damage; RDI, root damage index; SP, soil population; PM, plant mortality; P. neg, *Pratylenchus neglectus*; C. xen, *Criconemoides xenoplax*; M. jav, *Meloidogyne javanica*; M. inc, *M. incognita*. Error bars represent 95% confidence intervals.

Within contained studies, nematode species was an important variable in determining intercropping impact, with *P. neglectus* and *C. xenoplax* reported as the most impacted (reduced focal crop damage) and *M. incognita* and *M. javanica* as the least affected species of those commonly studied (Fig. 3). The method by which damage, or impact, was assessed is also a significant variable in contained studies. Those studies that assessed roots, either via directly measuring root damage or assessing damage against a root damage index (RDI) showed substantially greater impacts from intercropping, while those that explored nonroot specific outcomes (e.g. nematode soil population or plant mortality) reported less impact. (Fig. 3).

**Fig. 4:**
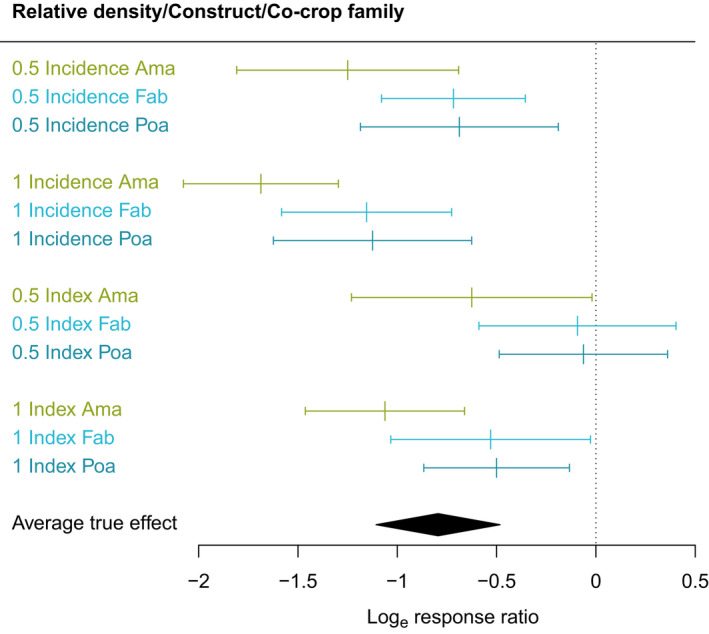
Predicted effects of intercropping on disease damage with combinations of the moderators retained in the final model for the DU1 dataset, showing relative density set to 0.5 or 1 and the co‐crop families most represented in the data (Ama, Amaryllidaceae; Fab, Fabaceae; Poa, Poaceae). Error bars represent 95% confidence intervals.

Disease control variables that directly and significantly influenced intercrop outcomes within in‐field, or uncontained, studies included intercrop family and focal crop density in the intercrop treatment relative to that in the monocrop control. Pathogen type was not a significant variable within the meta‐analysis, suggesting that pathogen type was much less important to intercropping yield outcomes compared with intercrop and focal crop density. Of the most studied intercrop families, Amaryllidaceae was more effective than either Poaceae or Fabaceae (Fig. 4). Methodologically, only the measurement construct was found to be significant in influencing perceived focal crop outcomes, with disease incidence indicative of greater impact relative to studies that utilised a disease index (Fig. 4). Contrary to that observed with nematode intercrop effects, neither disease type nor field fertilisation significantly influenced intercrop reduction of disease damage to focal crop.

The overall summary response ratio estimates for the full uncontained (NU1) and contained (NC1) nematode datasets were quite close, 0.60 and 0.55, respectively (Table S3), suggesting that experiments in pots may slightly overestimate the effectiveness of intercropping against nematodes. For the full uncontained (DU1) and contained (DC1) disease datasets, however, the overall summary true effect response ratio estimates were 0.45 and 0.56, respectively (Table S3), indicating that experiments in pots substantively underestimated the effectiveness of intercropping against soil‐borne diseases.

### Impacts of publication bias on model outcomes

Publication bias towards positive outcomes does not appear to have had a significant effect on our results: the fail‐safe numbers for the NU1, NC2, and DU1 datasets were 2250, 9773, and 3513, respectively, and no bias in the datasets was detected in the funnel plots (Figs S1–S3).

## Discussion

We found strong quantitative evidence in support of intercropping as an effective method of control for both plant‐parasitic nematodes and soil‐borne diseases.

Nematode impacts were reduced by 40% in‐field experiments, and 45% in pot studies (Table S3). The meta‐regression analysing the effects of intercropping on focal crop yield showed that anything below a 15% increase in nematode impacts was associated with an equal or greater yield in the intercropped fields (Fig. 1). This counterintuitive result suggests that, in intercropping systems, moderate increases in nematode damage are disconnected from yield outcomes. This indicates that other mechanisms arising from associational resistance may benefit yield, not solely pest reduction. However, the calculated regression line has substantial confidence intervals for both slope and intercept (Fig. 1) creating possible outcomes that range from (1) effectively no impact on yield outcomes in intercrop systems, (2) slightly reduced focal crop outcomes where intercrops are used, or (3) an even greater disconnect between nematode damage and focal crop yield in intercrop systems. Disease outcomes were reduced significantly through intercropping, by 55% across uncontained field studies but only 44% when experiments in pots were analysed (Table S3).

### Drivers of variability in outcomes

Variability in the effectiveness of intercropping as a control strategy is driven by substantially different factors for nematodes compared with disease. Both nematode and pathogenic microbial survival are determined by soil texture, moisture, temperature and pH (Menzies, 1963; Wallace, 1966). On species‐specific bases both are also affected by plant released volatiles (van Agtmaal *et al*., 2018; de Boer *et al*., 2019; Sikder & Vestergård, 2020). It seems likely that the differences in field outcomes between the two originate from the capacity of nematodes to orient and move towards host species based on volatile cues (Turnbull *et al*., 2001; Turlings *et al*., 2012; Yang & van Elsas, 2018). Meta‐regression of published outcomes reveals that effective nematode control is determined primarily by nematode species in contained studies, or by intercrop family and soil nutrient status (whether the experiment incorporated fertilisation) in uncontained experiments. By contrast, the only variables that significantly influenced disease outcomes (and only within uncontained studies) included intercrop family and density of focal crop planting in the intercrop relative to that in the focal monocrop.

#### Intercrop family

Intercrop family explained a significant fraction of the efficacy of intercropping against nematodes in uncontained field studies. For example, interplanting with Asteraceae, Pedaliaceae or Solanaceae species was associated with better nematode control, whereas Poaceae, Brassicaceae and Fabaceae intercrops were associated with less effective control. Intercrop root exudates can lead to associational resistance to nematodes through several mechanisms: (1) acting as biocidal compounds, so killing pests, or reducing their fitness; (2) acting as an attractant, so trapping pests away from the focal crop; (3) acting as a repellent, reducing pest population near the focal crop; or (4) modifying hatching rates, creating phenological mismatch between crops and early‐stage feeding (Sikder & Vestergård, 2020). Biocidal effects can include the housing of organisms such as nematode‐deterring endoroot bacteria, or, as for the Brassicaceae, the production of biofumigant compounds such as glucosinolates, which are metabolic precursors to bioactive isothiocyanates (Brennan *et al*., 2020). It is interesting to note the limited efficacy of the Brassicaceae in this meta‐analysis, despite their production of glucosinolates and their frequent use as biofumigants. Nematodes have been shown to be attracted to plant root volatiles (Rasmann *et al*., 2005; Turlings *et al*., 2012), and trap crops, including Asteraceae, have been shown to be effective in this context through a combination of lack of susceptibility and reduction of nematode hatch (Tsay *et al*., 2004).

Examples in which exudates can operate by combinations of these mechanisms are common, making these intercrops particularly effective. For example, lauric acid, exuded from *Chrysanthemum coronarium* roots, has been shown to be both attractive and lethal to *M. incognita* second‐stage juveniles, reducing galling on intercropped tomato plants (Dong *et al*., 2014). Some root exudates may accumulate, or persist at residual concentrations, after intercropping. Microbial parasites or allelochemicals may still be effective in reducing nematode numbers or effect, even at lower concentrations and could provide protection after intercrop removal (Mohan *et al*., 2020; Sikder & Vestergård, 2020).

Many intercrops produce nematocidal root exudates but, even when these compounds are not actively exuded from roots, nematodes may still be exposed to these allelochemicals when feeding upon, or penetrating, intercrop roots or encountering allelochemicals in the soil after root degradation. Examples include root tissues and exudates from Asteraceae family species that have been shown to contain compounds with nematocidal properties (Sánchez De Viala *et al*., 1998; Mohney *et al*., 2009; Weidenhamer *et al*., 2009), at levels sufficient to reduce nematode damage and reproduction (Salem & Osman, 1988) and are lethal and inhibit egg hatching (Siddiqui & Alam, 1987; Tsay *et al*., 2004).

Many plants produce root exudates that are not toxic but act as sensory stimulants, attracting or repulsing nematodes. For example, Solanaceae treatments were more effective than nearly all other intercrop families in nematode control (Fig. 2). Solanaceae may be particularly effective against nematodes because they emit methyl salicylate (MSA) (Murungi *et al*., 2018). MSA has been shown to be an effective attractant for *M. incognita* (Wuyts *et al*., 2006) and also acts a phytohormone, released during herbivory (Lin *et al*., 2017). Furthermore, exposure to MSA can confer resistance to agricultural pests (Bar‐Nun & Mayer, 2008) and its metabolism regulates plant defence signalling and systemic acquired resistance in exposed plants (Chen *et al*., 2019). Solanaceae therefore appears to confer multiple beneficial outcomes that build upon MSA attraction to nematodes, signalling other plants to prime defences and nematocidal action. Another example of a multi‐impact signalling effect can be found in intercropping with brassicaceous species, currently used as trap crops in rotations with sugar beet. *Brassica* spp. are beneficial intercrops, as their roots are attractive to, and can be invaded by, sugar beet cyst nematodes, but the sex ratio of the subsequent generation is heavily skewed towards males, leading to population decline (Caubel & Chaubet, 1985; Lelivelt & Hoogendoorn, 1993; Ratnadass *et al*., 2012).

#### Impacts of changing focal crop planting density

Whereas intercrop family was an important factor in determining disease reduction in uncontained studies, effects from interplanting on soil‐borne disease were greater when the density of focal crop plants in the intercrop treatment were kept closer to densities in focal monocrop control. This may be evidence of the ‘dilution effect’, whereby disease reduction is achieved by decreasing the frequency of hosts rather than absolute density (Civitello *et al*., 2015). Interplanting maize at a spacing of 5 cm decreased red crown rot in soybean more than a spacing of 20 cm (Gao *et al*., 2014). They suggested that greater density of maize roots reduced root‐to‐root transmission of the pathogen, in this case due to higher planting density rather than enhanced root growth.

Dispersal of pathogens across the crop system is a key determinant of disease incidence and spread (Tack *et al*., 2014). Therefore, interception of propagules travelling between roots could be an effective intercropping mechanism, leading to a lower incidence of disease and a process shown to be effective against aerial pathogens (Bouws & Finckh, 2008; Fernández‐Aparicio *et al*., 2011). Size and location of intercrop root systems are important in reducing disease incidence. For example, bacterial wilt was only reduced when cowpea was planted between tomato within rows, not when it was planted between the rows of tomato, or when Welsh onion (which had smaller root systems) was interplanted with tomato (Michel *et al*., 1997).

Greater plant density also causes shading (reducing soil temperatures) and greater evapotranspiration (reducing soil moisture), producing less favourable conditions for infection and disease development (Robinson *et al*., 1987; Olasantan, 1988; Yadav & Lalramliana, 2012). Retention of focal crop density could make intercropping more attractive to growers, as yield per unit area could be maintained while also reducing disease incidence.

#### Susceptibility of nematode species

Intercropping treatment impact in contained experiments was significantly influenced by the species of nematode involved. Contained studies are likely to restrain nematode mobility, enforcing the interaction with nonvolatile root exudates that may not travel far from the root or diffuse volatiles concentrated within contained soils (Erb *et al*., 2013). Studies on nematode susceptibility to root metabolites show substantial variation in their responses, only partially attributable to nematode lifestyle. Such species‐dependent resistance to repelling and/or toxic compounds explains the key role of nematode species identity in outcomes (Sikder & Vestergård, 2020).

Perhaps surprisingly, neither host status nor susceptibility of intercrop species to nematode attack was retained as significant in any of the meta‐regressions in which they were included, suggesting that disrupting nematode movement by adding a nonhost as intercrop to reduce nematode damage is ineffective. However, information on host status/susceptibility was unavailable for nearly half the experiments included in the full nematode dataset, limiting the scope of this result. The unimportance of intercrop species' host status or susceptibility level is positive from an agronomic point of view, as finding suitable nonhost species for intercropping might be challenging, especially for pests with wide host ranges such as the root knot nematode, *Meloidogyne incognita*. Host status/susceptibility may be important in intercrop impacts on soil‐borne disease transmission, but we were unable to assess this.

#### Field fertilisation

Soil and plant nutrient status are key drivers of ecological interactions between plants and their natural enemies (Holopainen *et al*., 1995). Associational susceptibility is often perceived to be driven by competition for nutrients between plants and reduced resource in focal crop plants. Here we found, perhaps surprisingly, that increasing resource availability through fertilisation reduced nematode control efficacy of intercropping in field experiments. There are several potential explanations for this surprising outcome. Plants can respond to nutrient (particularly N/P) stress by increasing root length/branching and releasing root exudates to access/forage for new soil nutrient pools (Khan *et al*., 2016). This increased root volume occupies a greater soil volume, leading to increased interception of pests by intercrop roots and/or enhanced exposure to allelopathic root exudates or root‐altered soil conditions. As with other types of plant defence (De Long *et al*., 2016), nutrient levels affect composition and quantity of root exudates. Nutrient‐rich plants have been shown to release fewer defensive volatiles (Fernandez‐Martinez *et al*., 2018). When maize was grown under phosphorus‐limiting conditions, their roots produced more cinnamic acid (with strong activity against the soybean red crown rot pathogen *Cylindrocladium parasiticum*) and more salicylic acid (inducing resistance against pathogens) (Gutjahr & Paszkowski, 2009; Gao *et al*., 2014). Plant nutrient status also creates indirect effects on pests: enhanced competition under reduced nutrients with a second plant species can cause morphological or physiological changes in a focal crop, such that it becomes a less suitable host (Trenbath, 1993).

### Conclusion

Intercropping can be an effective tool for reducing nematode damage and disease in agricultural fields, with averaged reductions in impacts on the performance of the focal crop of 40% and 55% respectively. Our study also showed that treatments in fields with nematode pests will improve when using reduced field fertilisation approaches, whereas diseased field outcomes will be improved with greater planting densities. Generally, across all intercropping systems the intercrop has a significant impact on outcomes, and further characterisation of specific modes of associational resistance derived for each intercrop/pest pairing is needed to provide confidence in focal crop yield outcomes.

Future research should avoid experiments with potted plants. Our analysis demonstrates that outcomes from contained experiments are not indicative of *in situ* outcomes or mechanisms of action. Similarly, although our results point to effective pest and disease control, it remains unclear whether even greater nematode control can be achieved through reduced, intermediate nutrient content in agricultural fields. Further in‐field research into intermediate fertilisation status is needed to clarify this question. Similarly, quantifying maximally effective planting densities will require substantially more research to understand whether these can be generalised across a range of agroecosystems, or whether they are crop/pathogen specific.

Overall, our study demonstrates the potential of associational resistance, via intercropping and other mechanisms, to reduce the impact of soil pests and disease on crops. It provides support to farming practices based on ecological approaches to deliver more sustainable productive agricultural systems.

## Author contributions

VGAC performed the literature search and the meta‐analyses. VGAC wrote the first draft of the manuscript, with equal editorial input from KRR and SEH after the first draft.

## Supporting information


**Fig. S1** Funnel plot analysis for the ‘Nematode Uncontained’ model 1 (Table S4).
**Fig. S2** Funnel plot analysis for the ‘Nematode Contained’ model 2 (Table S4).
**Fig. S3** Funnel plot analysis for the ‘Disease Uncontained’ model 1 (Table S4).
**Notes S1** Search query strings.
**Table S1** Moderator variables, with categories/ranges and number of experiments that reported them.
**Table S2** Sources of information on currently accepted binomials, plant host status and susceptibility, and standard agronomic practices.
**Table S3** Summary meta‐analyses results.
**Table S4** Name, size, initial and (where reached) final model specifications, and moderator omnibus test results for each meta‐regression performed using the nematode and disease data.
Please note: Wiley Blackwell are not responsible for the content or functionality of any Supporting Information supplied by the authors. Any queries (other than missing material) should be directed to the *New Phytologist* Central Office.

## Data Availability

Data sharing is not applicable to this article as no new data were created or analysed in this study. All materials obtained through systematic review and which supports the meta‐analysis findings of this study are described in the Supplementary Material of this article.
