# Supplementary material for: Associational resistance through intercropping reduces yield losses to soil‐borne pests and diseases
Source: New Phytol. 2022 Jul 1;235(6):2393–405. doi: 10.1111/nph.18302 (PMC9545407; doi:10.1111/nph.18302)

New Phytologist Supporting Information

Article Title: Associational resistance through intercropping reduces yield losses to soil borne pests and diseases.

Authors: Victoria G. A. Chadfield, Sue E. Hartley, and Kelly R. Redeker

Article acceptance date: May 4th, 2022

Table S1: Moderator variables, with categories/ranges and number of experiments which reported them. # indicates that when moderator status was not explicitly stated, the negative option was assumed, e.g. not irrigated, unfertilised, etc. Where figure is in parentheses, that moderator was not tested in any of the models run for that data subset. Square (or {}) brackets indicate that testing was not possible as all (or almost all) values for that moderator were the same for that subset of the data. Where NA appears, it indicates the field is 'not applicable'.

		No. of uncontained/contained experiments reporting moderator data	
Moderator	Categories/range	Nematode damage	Disease incidence
Measurement construct	Plant mortality;<br>Reproduction ratio soil;<br>Root damage;<br>Root damage index;<br>Root population;<br>Soil population;<br>Disease incidence;<br>Disease index	138/188	89/28
Co-crop family	Amaryllidaceae; Apiaceae;<br>Apocynaceae; Asparagaceae;<br>Asteraceae; Brassicaceae;<br>Convolvulaceae; Cucurbitaceae;<br>Euphorbiaceae; Fabaceae;<br>Geraniaceae;<br>Lamiaceae; Linaceae;<br>Meliaceae;<br>Moringaceae; Pedaliaceae;<br>Plantaginaceae; Poaceae;<br>Rosaceae;<br>Solanaceae; Violaceae;<br>Zingiberaceae	138/188	89/28
Conditions	Field; greenhouse	138/[188]	(89)/[28]
Inoculation timing (timing of inoculum addition relative to focal crop sowing/transplanting)	Present; After	[138]/188	(89)/(28)

		No. of uncontained/contained experiments reporting moderator data	
Moderator	Categories/range	Nematode damage	Disease incidence
Water status#	Irrigated; not irrigated	138/[188]	(89)/[28]
Sterilisation status# (if growing medium was sterilised or not)	Sterilised; unsterilised	[138]/188	[89]/28
Fertilisation status#	Fertilised; unfertilised	138/188	89/(28)
Inoculum type	Artificial; natural	[138]/{188}	89/{28}
Nematode species	*Criconemoides xenoplax*; *Meloidogyne hapla*; *M. incognita*; *M. javanica*; *Pratylenchus alleni*; *P. brachyurus*; *P. coffeae*; *P. neglectus*; *P. penetrans*; *P. zeae*; *Radopholus similis*; *Rotylenchulus reniformis*; *Tylenchorhynchus brassicae*; *T. mashhoodi*; *T. clarus*; *Tylenchulus semipenetrans*; *Xiphinema americanum*	112/185	NA
Nematode lifestyle	Endoparasite; ectoparasite	127/188	NA
Pathogen species	Calonectria ilicicola; Fusarium oxysporum; F. solani; Gaeumannomyces graminis; Macrophomina phaseolina; Phytophthora capsici; Ralstonia solanacearum; Rhizoctonia solani; Streptomyces scabiei; Verticillium dahliae	NA	67/27
Pathogen genus	Calonectria; Fusarium; Gaeumannomyces;Macrophomina; Phytophthora; Pythium; Ralstonia; Rhizoctonia; Streptomyces;	NA	81/28

		No. of uncontained/contained experiments reporting moderator data	
Moderator	Categories/range	Nematode damage	Disease incidence
Relative density (focal crop density in the intercrop treatment as a proportion of its density in the monocrop control)	0.125 to 1	137/180	89/(28)
Co-crop susceptibility	Immune; highly resistant; resistant; moderately resistant; moderately susceptible; susceptible	175/151	NA
Co-crop host status	Host; non-host	86/153	NA
Yield measurement construct	Per unit area;<br>Per plant	76	66

Table S2: Sources of information on currently accepted binomials, plant host status and susceptibility, and standard agronomic practices

Information	Source reference
Pathogen species accepted binomials	Roskov Y, Abucay L, Orrell T, Nicolson D, Kunze T, Culham A, Bailly N, Kirk P, Bourgoin T, DeWalt RE, Decock W, De Wever A. eds. 2014. Species 2000 & ITIS Catalogue of Life. Digital resource at www.catalogueoflife.org/col (accessed 29th October 2014).
Nematode species accepted binomials	Bongers AMT. 2014. *Tylenchorhynchus brassicae* Siddiqi, 1961. Fauna Europaea, <https://fauna-eu.org> (accessed 14/11/2014)<br><br>Ferris H. 1999a. Nemabase search engine for the host status of plants to nematodes (Revised 13/07/2012) <http://plpnemweb.ucdavis.edu/nemaplex/Nemabase2010/NemabasePlantQuery.aspx> (Accessed 02/01/2013)

Plant susceptibility and host status to nematodes

Adegbite AA, Adesiyan SO, Agbaje GO, Omoloye AA. 2005. Host suitability of crops under yam intercrop to root-knot nematode (*Meloidogyne incognita* Race 2) in south-western Nigeria. *J. Agric. Rural Dev. Trop. Subtrop.* 106: 113–118.

Alam MM, Saxena SK, Khan AM. 1977. Influence of interculture of marigold and margosa with some vegetable crops on plant growth and nematode population. *Acta Bot. Indica* 5: 33–39.

Arim OJ, Waceke JW, Waudo SW, Kimenju JW. 2006. Effects of *Canavalia ensiformis* and *Mucuna pruriens* intercrops on *Pratylenchus zeae* damage and yield of maize in subsistence agriculture. *Plant Soil* 284: 243–251. doi:10.1007/s11104-006-0053-9

Curto G, Dallavalle E, Lazzeri L. 2005. Life cycle duration of *Meloidogyne incognita* and host status of Brassicaceae and Capparaceae selected for glucosinate content. *Nematology* 7: 203–212. doi:10.1163/1568541054879494

El-Hamawi MH, Youssef MMA, Zawam HS. 2004. Management of *Meloidogyne incognita*, the root-knot nematode, on soybean as affected by marigold and sea ambrosia (damsisa) plants. *J. Pest Sci.* 77: 95–98. doi:10.1007/s10340-003-0034-1

Ferris H. 1999a. Nemabase search engine for the host status of plants to nematodes (Revised 13/07/2012) <http://plpnemweb.ucdavis.edu/nemaplex/Nemabase2010/NemabasePlantQuery.aspx> (Accessed 02/01/2013)

Germani G, Plenchette C. 2004. Potential of *Crotalaria* species as green manure crops for the management of pathogenic nematodes and beneficial mycorrhizal fungi. *Plant Soil* 266: 333–342. doi: 10.1007/s11104-005-2281-9

Mashela PW, Pofu KM. 2017. Nematode resistance to tropical *Meloidogyne* species in *Moringa oleifera*. *Research on Crops* 18: 513–517. doi:10.5958/2348-7542.2017.00088.2

McSorley R. 1999. Host suitability of potential cover crops for root-knot nematodes. *J. Nematol.* 31: 619–623.

Meyer JR, Zehr EI, Meagher Jr RL, Salvo SK. 1992. Survival and growth of peach trees and pest populations in orchard plots managed with experimental ground covers. *Agric. Ecosyst. Environ.* 41: 353–363. doi: http://dx.doi.org/10.1016/0167-8809(92)90121-Q

Shigaki T, Gray FA, Delaney RH, Koch DW. 1998. Evaluation of host resistance and intercropping for management of the northern root-knot nematode in sainfoin, *Onobrychis viciifolia*. *J. Sustain. Agric.* 12: 23–39. doi:10.1300/J064v12n01_04

Timper P, Davis RF, Tillman PG. 2006. Reproduction of *Meloidogyne incognita* on winter cover crops used in cotton production. *J. Nematol.* 38: 83–9.

Tsay TT, Wu ST, Lin YY. 2004. Evaluation of Asteraceae plants for control of *Meloidogyne incognita*. *J. Nematol.* 36: 36–41.

Zehr EI, Lewis SA, Bonner MJ. 1986. Some herbaceous hosts of the ring nematode (*Criconemella xenoplax*). *Plant Dis.* 70: 1066–1069.

Information	Source reference
	Zehr EI, Aitken JB, Scott JM, Meyer JR. 1990. Additional hosts for the ring nematode, *Criconemella xenoplax*. *J. Nematol.* 22: 86–89.
Nematode lifestyle	Ferris H. 1999a. Nemabase search engine for the host status of plants to nematodes (Revised 13/07/2012) <http://plpnemweb.ucdavis.edu/nemaplex/Nemabase2010/NemabasePlantQuery.aspx> (Accessed 02/01/2013)
Recommended agronomic practice for lentils (as details not reported in Adbel-Monaim & Abo-Elyousr (2012) and authors unavailable)	Egyptian Ministry of Agriculture (pers. comm.)

Table S3: Summary meta-analyses results (CI = confidence interval; *** indicates *p*-value<0.001, ** indicates *p*-value<0.01, * indicates *p*-value<0.05)

Data subset name	No. of experiments (*k*)	Estimated average true effect size as response ratio (95% CI)
Nematode Uncontained 1 (NU1)	138	0.60 (0.45 to 0.80) ***
Nematode Uncontained 2 (NU2)	137	0.59 (0.45 to 0.79) ***
Nematode Uncontained 3 (NU3)	127	0.59 (0.45 to 0.79) ***
Nematode Uncontained 4 (NU4)	114	0.66 (0.51 to 0.86) **
Nematode Uncontained 5 (NU5)	79	0.67 (0.50 to 0.91)**
Nematode Uncontained 6 (NU6)	68	0.69 (0.50 to 0.95) *
Nematode Contained 1 (NC1)	188	0.55 (0.44 to 0.69) ***
Nematode Contained 2 (NC2)	185	0.58 (0.46 to 0.72) ***
Nematode Contained 3 (NC3)	180	0.55 (0.43 to 0.70) ***
Nematode Contained 4 (NC4)	153	0.62 (0.49 to 0.78) ***
Nematode Contained 5 (NC5)	151	0.62 (0.49 to 0.78) ***
Disease Uncontained 1 (DU1)	89	0.45 (0.33 to 0.62) ***
Disease Uncontained 2 (DU2)	81	0.44 (0.32 to 0.62) ***
Disease Uncontained 3 (DU3)	67	0.43 (0.29 to 0.64) ***
Disease Contained 1 (DC1)	28	0.56 (0.43 to 0.72) ***
Disease Contained 2 (DC2)	27	0.56 (0.43 to 0.73) ***

Table S4: Name, size, initial and (where reached) final model specifications, and moderator omnibus test results for each meta-regression performed using the nematode and disease data (CI = confidence interval; '***' indicates *p*-value<0.001, '**' indicates *p*-value<0.01, '*' indicates *p*-value<0.05, ' ' indicates *p*-value>0.05). Where NA appears, it indicates that the unique moderator for that data subset was not retained and so model reduction was not continued.

Data subset	Moderators included in initial full model	Moderators retained in final model	Final omnibus test of moderators ($Q_{M\ (d.f.)}$)
NU1	Measurement construct + Co-crop family + Conditions + Water status + Fertilisation status	Co-crop family + Fertilisation status	$61.1336_{(13)}$ ***
NU2	Measurement construct + Co-crop family + Conditions + Water status + Fertilisation status + Relative density	NA	NA
NU3	Measurement construct + Co-crop family + Conditions + Water status + Fertilisation status + Nematode lifestyle	NA	NA
NU4	Measurement construct + Co-crop family + Conditions + Water status + Fertilisation status + Nematode sp.	NA	NA
NU5	Measurement construct + Co-crop family + Conditions + Water status + Fertilisation status + Co-crop host status + Relative density	NA	NA
NU6	Measurement construct + Co-crop family + Conditions + Water status + Fertilisation status + Co-crop susceptibility + Co-crop host status + Relative density	NA	NA
NC1	Measurement construct + Co-crop family + Sterilisation status + Fertilisation status + Nematode lifestyle + Inoculation timing	Measurement construct	$8.7734_{(5)}$
NC2	Measurement construct + Co-crop family + Sterilisation status + Fertilisation status + Nematode lifestyle + Inoculation timing + Nematode sp.	Measurement construct + Nematode sp.	$32.4811_{(15)}$ **

Data subset	Moderators included in initial full model	Moderators retained in final model	Final omnibus test of moderators ($Q_{M\ (d.f.)}$)
NC3	Measurement construct + Co-crop family + Sterilisation status + Fertilisation status + Nematode lifestyle + Inoculation timing + Relative density	NA	NA
NC4	Measurement construct + Co-crop family + Sterilisation status + Fertilisation status + Nematode lifestyle + Inoculation timing + Co-crop host status + Nematode sp.	NA	NA
NC5	Measurement construct + Co-crop family + Sterilisation status + Fertilisation status + Nematode lifestyle + Inoculation timing + Co-crop susceptibility + Co-crop host status + Nematode sp.	NA	NA
DU1	Measurement construct + Co-crop family + Fertilisation status + Relative density + Inoculum type	Measurement construct + Co-crop family + Relative density	$42.4002_{(11)}$ ***
DU2	Measurement construct + Co-crop family + Fertilisation status + Relative density + Inoculum type + Pathogen genus	NA	NA
DU3	Measurement construct + Co-crop family + Fertilisation status + Relative density + Inoculum type + Pathogen species	NA	NA
DC1	Measurement construct + Co-crop family + Pathogen genus + Sterilisation status	None	NA
DC2	Measurement construct + Co-crop family + Pathogen species + Sterilisation status	NA	NA
Nematode Yield	Yield measurement construct + $L_{nematode}$	$L_{nematode}$	$16.2450_{(1)}$ ***
Disease Yield	Yield measurement construct + $L_{disease}$	NA	NA

Notes S1 – Search query strings

Web of Science

Topic=(intercrop* OR "inter-crop*" OR cocrop* OR "co-crop*" OR (mixed NEAR/1 crop*) OR interplant* OR "inter-plant*" OR bicrop* OR "bi-crop" OR polycultur* OR "poly-cultur*" OR dicultur* OR "di-cultur*" OR (cover crop*) or companion*) AND

Topic=(disease* OR patho* OR nemat*)

Timespan=All Years

Databases=SCI-EXPANDED, CPCI-S

Lemmatization=On

Trove

One term at a time from the full list (intercrop* OR "inter-crop*" OR cocrop* OR "co-crop*" OR (mixed crop*) OR interplant* OR "inter-plant*" OR bicrop* OR "bi-crop" OR polycultur* OR "poly-cultur*" OR dicultur* OR "di-cultur*" OR (cover crop*) or companion*) in the first search box and the three other search terms in the second search box.

So for example:

Keyword= intercrop* AND

Keyword=(nemat* OR disease* OR pathogen*)

Format=Thesis, Conference Proceedings

Language=English

British Library EThOS

One term at a time from the full list (intercrop* OR "inter-crop*" OR cocrop* OR "co-crop*" OR (mixed crop*) OR interplant* OR "inter-plant*" OR bicrop* OR "bi-crop" OR polycultur* OR "poly-cultur*" OR dicultur* OR "di-cultur*" OR (cover crop*) or companion*) in the first search box and the three other search terms in the second search box.

So for example:

Keywords= intercrop* AND

Keywords=(nemat* OR disease* OR pathogen*)

Indian Citation Index

Topic=Intercrop* AND

Topic=disease* OR patho* OR nemat*

Timespan=All Years

Figure S1: Funnel plot analysis for the "Nematode Uncontained" model 1 (Table S4).

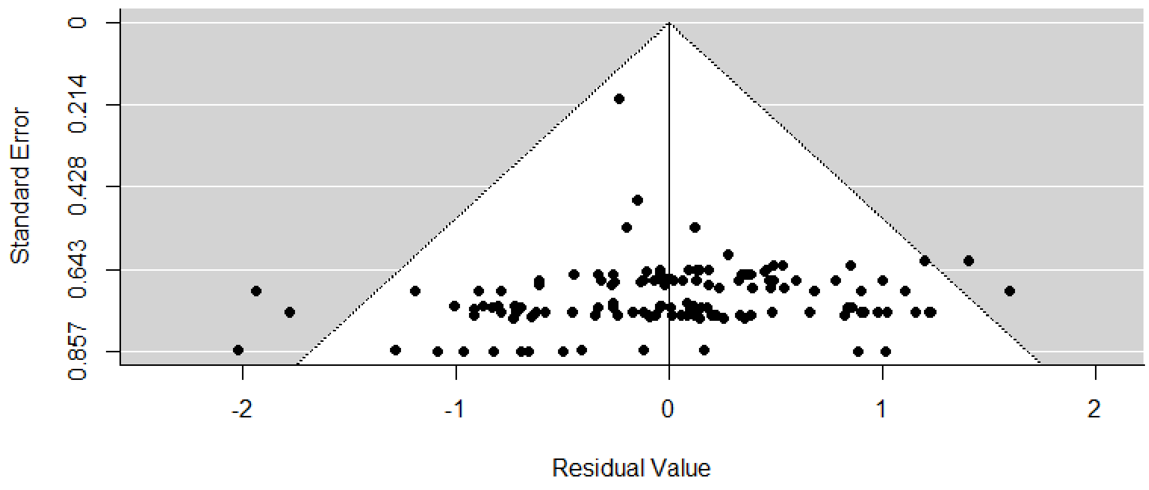

Figure S2: Funnel plot analysis for the "Nematode Contained" model 2 (Table S4).

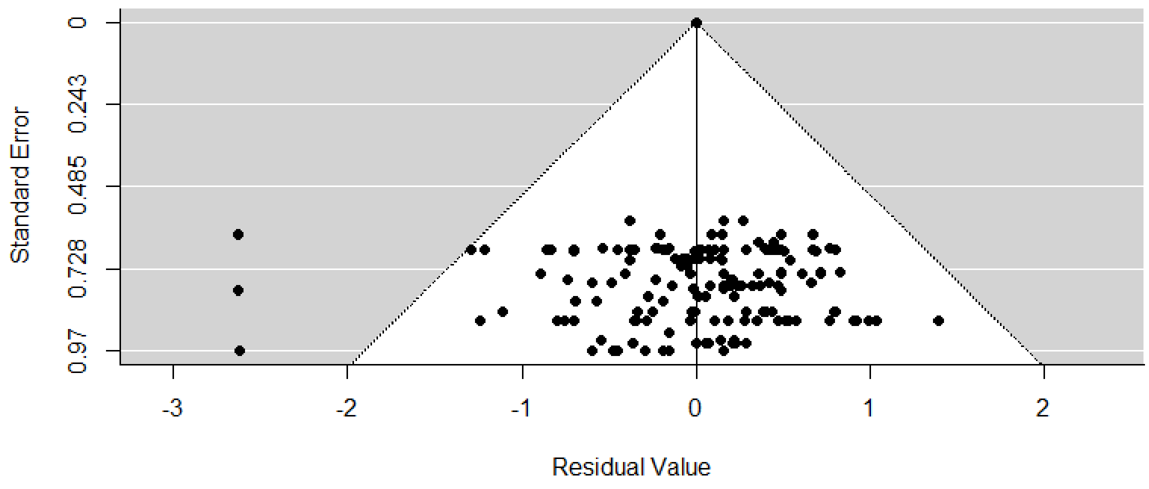

Figure S3: Funnel plot analysis for the "Disease Uncontained" model 1 (Table S4).

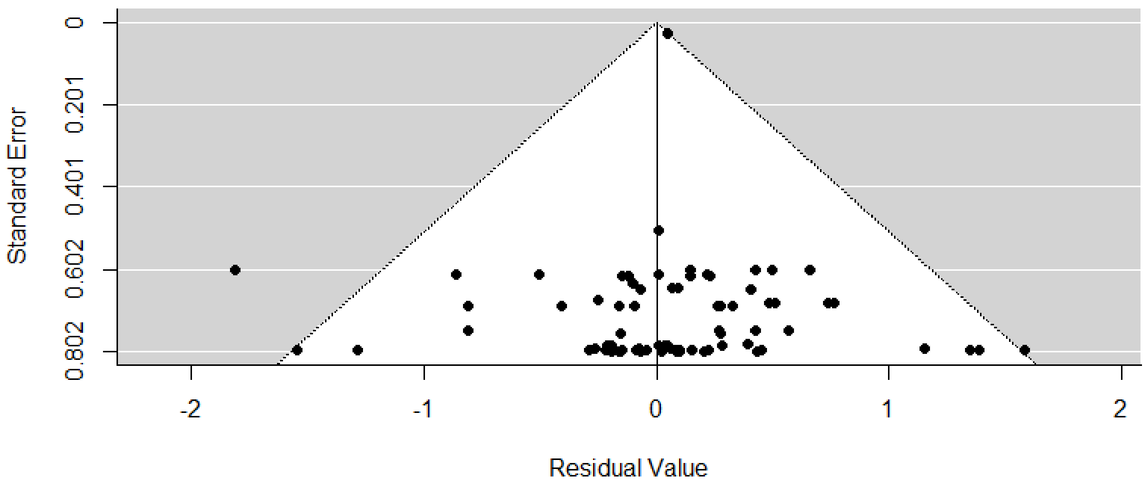

Supplement: Supplementary file 1 — Fig. S1 Funnel plot analysis for the ‘Nematode Uncontained’ model 1 (Table S4). Fig. S2 Funnel plot analysis for the ‘Nematode Contained’ model 2 (Table S4). Fig. S3 Funnel plot analysis for the ‘Disease Uncontained’ model 1 (Table S4). Notes S1 Search query strings. Table S1 Moderator variables, with categories/ranges and number of experiments that reported them. Table S2 Sources of information on currently accepted binomials, plant host status and susceptibility, and standard agronomic practices. Table S3 Summary meta‐analyses results. Table S4 Name, size, initial and (where reached) final model specifications, and moderator omnibus test results for each meta‐regression performed using the nematode and disease data. Please note: Wiley Blackwell are not responsible for the content or functionality of any Supporting Information supplied by the authors. Any queries (other than missing material) should be directed to the New Phytologist Central Office. [file NPH-235-2393-s001.pdf]
